# Membrane orientation regulates GAPR-1 oligomerization that is suitable for interaction with beclin 1

**DOI:** 10.1007/s00018-026-06329-4

**Published:** 2026-07-28

**Authors:** Ziying Shen, Robin A. Corey, Hidde van Doorne, Iris Verhoek, Renée Koopman, Daniel S. Huitema, Samiksha Sardana, Marc P. Baggelaar, Phillip J. Stansfeld, Dora V. Kaloyanova, J. Bernd Helms

**Affiliations:** 1 Division of Cell Biology, Metabolism and Cancer, Faculty of Veterinary Medicine, Utrecht University, Utrecht, The Netherlands; 2 School of Physiology, Pharmacology & Neuroscience, Biomedical Sciences Building University Walk, Bristol, BS8 1TD UK; 3 Protein Research Centre, Department of Chemistry, Faculty of Science, Utrecht University, Utrecht, The Netherlands; 4 Biomolecular Mass Spectrometry & Proteomics, Faculty of Science, Utrecht University, Utrecht, The Netherlands; 5 School of Life Sciences, Department of Chemistry, University of Warwick, Coventry, CV4 7AL UK; 6 Department of Laboratory Animal Center, General Hospital of Northern Theater Command, 83 Wenhua Road, Shenhe District, Shenyang, Liaoning 110016 China

**Keywords:** CAP superfamily of proteins, Protein-lipid interactions, Protein-protein interactions, Protein oligomerization, N-myristoylation, Autophagy

## Abstract

Golgi-associated plant pathogenesis-related protein 1 (GAPR-1) is a beclin 1-interacting peripheral membrane protein that is anchored to the Golgi complex via an N-myristoyl-group and ionic interactions. Here we investigated the determinants of GAPR-1 membrane binding using molecular dynamics (MD) simulations and applied the results to investigate the subsequent oligomerization into biomolecular condensates and interaction with beclin 1. MD simulations revealed a specific orientation of GAPR-1 upon docking to a negatively charged lipid bilayer involving lysines at position 7 and 33 (Lys7 and Lys33). Substitution of these lysines with glutamine changed the preferred membrane orientation of GAPR-1. Upon deletion of the myristoyl-group, GAPR-1 remained docked to the lipid bilayer, but lost its preferred orientation, suggesting that myristoylation constrained the dynamics of the protein at the membrane surface. Upon mutation of Lys7 to glutamine and expression in *Saccharomyces cerevisiae*, [K7Q]GAPR-1 was prevented from myristoylation and condensate formation was severely impaired. Similar results were obtained when N-terminal myristoylation of GAPR-1 was prevented by mutating amino acid Gly2. However, in contrast to [G2A]GAPR-1, [K7Q]GAPR-1 could not interact with beclin 1 anymore upon co-expression in yeast cells, and failed to interfere with beclin 1-dependent autophagy in human cells. Our combined data show that GAPR-1 requires a defined orientation at the membrane to control protein homo-and hetero-oligomerization and to execute its function. We revealed a novel function of protein myristoylation in which it determines the motional freedom of specific protein orientations, which may have relevance for the entire myristoylated proteome.

**Supplementary Information:**

The online version contains supplementary material available at https://doi.org/10.1007/s00018-026-06329-4.

## Introduction

Golgi-associated plant pathogenesis-related protein 1 (GAPR-1) is a peripheral membrane protein that belongs to the CAP superfamily, named after its founding members cysteine-rich secretory proteins (CRISPs), antigen 5 (Ag5), and pathogenesis-related 1 (PR-1). Proteins in this family contain a characteristic CAP domain that exhibits four highly conserved signature motifs and has a unique α-β-α sandwich fold structure [1–3]. Additional domains, mostly at the C-terminus of the CAP domain, define the different family members and contribute to evolutionary diversity. The role of the CAP domain and its conserved motifs, however, remains elusive. GAPR-1 is the only mammalian CAP protein without a signal sequence and without an additional extension at the C-terminus [4, 5]. Investigating the structure and function of GAPR-1 may shed light on the nature of the CAP domain.

GAPR-1, also known as GLIPR2, is a small peripheral membrane protein (17 kDa) with a positive charge (pI 9.4) and a myristoyl-group at its N-terminus [4]. It is predominantly found in lipid-enriched microdomains of the Golgi complex. GAPR-1 has been identified as a negative regulator of autophagy by binding to beclin 1 and retaining it at the cytosolic leaflet of the Golgi complex [6, 7]. GAPR-1 at Golgi membranes partially exists as a dimer. It was shown that GAPR-1 binds to negatively charged lipids and that membrane-binding to negatively charged liposomes in vitro also induces dimerization [8]. Prolonged incubation of GAPR-1 containing liposomes leads to the formation of oligomers and amyloid-like fibrils [9]. The amyloid-like aggregation of GAPR-1 is influenced by divalent metal-ions (Zn^2+^ and Cu^2+^) and a redox balance [10, 11].

Many amyloid-forming proteins can form dynamic liquid condensates before transitioning into solid-like fibrillar aggregates [12–15]. These reversible, liquid-like protein droplets have gained considerable interest as regulators of numerous biological processes. We previously demonstrated that GAPR-1 can form dynamic and reversible condensates using the *Saccharomyces cerevisiae* yeast model system [16]. GAPR-1 condensate formation in yeast is facilitated by membrane association of GAPR-1 [16], most likely by providing a platform for locally enhanced protein concentrations that can act as nucleation sites [17]. Other key factors, including protein post-translational modifications (PTMs), protein-protein interactions, and interaction with metal ions, can control the formation of these condensates [12, 17, 18]. Indeed, we previously showed that GAPR-1 condensate formation is e.g. sensitive to zinc ions [16]. Interestingly, upon co-overexpression of GAPR-1 and human beclin 1, they interfered with their mutual condensate formation [19]. This interference was based on the same type of protein-protein interactions as described for their in vivo interaction at Golgi membranes [6, 7].

In this study we combined a variety of methods, ranging from computational molecular simulations, in vitro biochemical and biophysical approaches, to in vivo yeast biomolecular condensate assays, to investigate the mechanisms underlying GAPR-1 association with membranes and its role in regulating protein oligomerization and condensate formation. Our findings reveal that the docking of GAPR-1 occurs in a unique orientation, initiating distinct GAPR-1 oligomerization and allowing interaction with beclin 1 and thus affecting autophagy. We discuss the role of the CAP domain in regulating protein oligomerization.

## Materials and methods

### Coarse-grained molecular dynamics simulations

Membrane binding of GAPR-1 was assayed using coarse-grained (CG) simulations. The monomeric GAPR-1 crystal structure was used as an input (Protein Data Bank ID code 4AIW [8]),. The inositol hexakisphosphate and all solvent atoms were removed. Protein atoms were converted to the CG Martini 3 force field [20] using the Martinize2 method [21]. Additional bonds of 500 kJ mol^− 1^ nm^− 2^ were applied between all protein backbone beads within 1 nm. Proteins were oriented randomly using the GROMACS *editconf* tool, with a different starting orientation for each repeat. Proteins were placed 8 nm from a membrane (centre-of-mass distance), which were built using the insane protocol [22]. See Table 1 for details of the membrane composition. Where used, phosphoinositide lipids were parameterised as described [23]. Simulation boxes were 10 × 10 × 20 nm^3^. All systems were solvated with Martini waters and Na^+^ and Cl^−^ ions to a neutral charge and at 150 mM concentration. Systems were minimized using the steepest descents method, followed by 1 ns equilibration with 5 fs time steps, then by 100 ns equilibration with 20 fs time steps, before 10 µs production simulations using 20 fs time steps, all in the NPT ensemble at 323 K with the V-rescale thermostat (τ_t_ = 1.0 ps) and semi-isotropic Parrinello-Rahman pressure coupling at 1 bar (τ_p_ = 12.0 ps). The Reaction-Field method was used to model long-range electrostatic interactions. Bond lengths were constrained to their equilibrium values using the LINCS algorithm. Simulations were run in GROMACS 2020.3 [24, 25]. Plots were made in Python using NumPy and Matplotlib, or made in Prism 10. MD snapshots were made using VMD [26]. Data were analysed using GROMACS tools.

**Table 1:** Simulations run in this study

System	Description	Runs
wtGAPR-1 with PC/PE/PS/PI4P membrane	CG	10 × 10 µs
wtGAPR-1 with PC/PE/PS/PI membrane	CG	10 × 10 µs
wtGAPR-1 with PC/PE/PS/PA membrane	CG	5 × 10 µs
wtGAPR-1 with myristate tail	AT	3 × 0.65 µs
wtGAPR-1 without myristate tail	AT	3 × 0.65 µs
[K7Q]GAPR-1 with myristate tail	AT	3 × 1.5 µs
[K7Q]GAPR-1 without myristate tail	AT	3 × 1.5 µs
[K33Q]GAPR-1 with myristate tail	AT	3 × 1.0 µs
[K33Q]GAPR-1 without myristate tail	AT	3 × 1.0 µs
Soluble wtGAPR-1 with myristate tail	AT	10 × 0.2 µs

### Atomistic MD simulations

Post CG-MD poses of GAPR-1 bound to the membrane were converted into atomistic poses using the CG2AT2 protocol [27]. Where included, mutations were added using the CG2AT2 protocol. Systems were described using the CHARMM36m force field [28] with TIP3P water. Where used, protein coordinates were processed in CHARMM-GUI [29] to add myristoylation to Gly2 of GAPR-1. These were then placed back into the membrane-bound pose, with the sole overlapping membrane lipid deleted. Alternatively, the GAPR-1 monomer containing myristoylation was built into an 8.2 × 8.2 × 8.2 nm^3^ solvated box with TIP3P water and Na^+^ and Cl^−^ ions to 150 mM. Systems were energy minimized using the steepest descents method, and subsequently equilibrated with positional restraints on heavy atoms for 1 ns in the NPT ensemble at 303 K with the V-rescale thermostat (τ_t_ = 1.0 ps) and semi-isotropic Parrinello-Rahman pressure coupling at 1 bar (τ_p_ = 5.0 ps), with a compressibility of 4.5 × 10^− 5^ bar ^− 1^. The Particle-Mesh-Ewald (PME) method was used to model long-range electrostatic interactions. Van der Waals (VdW) interactions were cut off at 1.2 nm. Bond lengths were constrained to the equilibrium values using the LINCS algorithm. Production simulations were run with 2 fs time step. Simulations were run in GROMACS 2020.3 [24, 25].

### Yeast strains, plasmids and culture media

Wild-type yeast strain W303 was transformed by Frozen-EZ yeast kit from Zymo research (Zymo Research, Orange, CA, USA) with a high copy (2µ) plasmid p423 carrying different constructs with C-terminally-tagged GFP (Table 2).

**Table 2:** Plasmids used in this study

Plasmid	Description	Source
p423-*GAL1*	*2 μm; HIS3; GAL1pr; CYC1term; AmpR*	[16]
pME3759	*p426-GAL1-GFP*	[16]
pNS 1000	*p423-GAL1-GAPR-1-GFP*	[16]
pNS 1001	*p423-GAL1-[G2A](ΔMyr)GAPR-1-GFP*	[16]
pBC 430	*p423-GAL1-[K7Q]GAPR-1-GFP*	This study
pBC 480	*p423-GAL1-[K33Q]GAPR-1-GFP*	This study
pBC 296	*p426-GAL1-GAPR-1-mCherry*	This study
pBC 490	*p426-GAL1-[K7Q]GAPR-1-mCherry*	This study
pBC 491	*p426-GAL1-[G2A](ΔMyr)GAPR-1-mCherry*	This study
pNS 400	*p423-GAL1-[C32S] GAPR-1-GFP*	From Dr. N. Sirati
pCW-7641	*pCW-MCS-Puro*	From Dr. J. Chang
pCW-7452	*pCW-MCS-BSD*	From Dr. J. Chang
pLenti-8619	*pLenti-CMV-GFP-Puro*	From Dr. J. Chang
pCW-10,228	*pCW-peredox-mCherry-BSD*	From Dr. J. Chang
pCW-8128	*wtGAPR-1-Myc-pCW-MCS-Puro*	This study
pCW- 8129	*K7QGAPR-1-Myc-pCW-MCS-Puro*	This study

All GAPR-1 mutants were constructed by using Phusion™ site-directed mutagenesis kit (Thermo Scientific, Massachusetts, USA) and p423-GAL1-GAPR-1-GFP [16] as template. The primers are listed in Table 3.

**Table 3:** Primers used in this study

Primer	Sequence
FW [K7Q]GAPR-1	5’-CAGCTTCCCAACAGTTTCATAATGAGGT-3’
REV [K7Q]GAPR-1	5’-CATTATGAAACTGTTGGGAAGCTGACT-3’
FW [K33Q]GAPR-1	5’-CACTGAAGCTCTGCCAGAACCTCAACCGG − 3’
REV [K33Q]GAPR-1	5’-CCGGTTGAGGTTCTGGCAGAGCTTCAGTG − 3’
FW [G2A]GAPR-1	5’-GATCCCCCATGGCCAAGTCAGCTTCCAAACAG-3’
REV [G2A]GAPR-1	5’-GCTGACTTGGCCATGGGGGATCCACTAGTTC-3’
FW GAPR-1	5’-GAACTAGTGGATCCCCCATGGGCAAGTCAGC-3’
REV GAPR-1 for mCherry	5’-CCTTGCTCACCATATCGATAAGCTTCTTCTTCGGCGGC-3’
FW mCherry for GAPR-1	5’-CCGCCGAAGAAGAAGCTTATCGATATGGTGAGCAAGGG-3’
REV mCherry	5’-GAATTCCTGCAGCCCCTACTTGTACAGCTCGTC-3’
FW wtGAPR-1-Myc	5’-GCGGTCGACATGGGCAAGTCAGCT-3’
REV wtGAPR-1-Myc	5’-GCGGGAATTCTTACAGATCCTCTTC-3’
FW GAPR-1-Myc[K7Q]	5’-CAGCTTCCCAACAGTTTCATAATG-3’
REV GAPR-1-Myc[K7Q]	5’-CATTATGAAACTGTTGGGAAGCTG-3’

Cells were pre-grown overnight in synthetic complete medium lacking the corresponding marker (SC-His/-Ura) and supplemented with 2% raffinose at 30 °C with orbital agitation (200 rpm) for 18 h (overnight). The next day, optical density at 600 nm (OD_600_ nm) was measured, and the cells were placed in selective SC medium (OD_600_=0.1) supplemented with 2% galactose to induce protein expression for indicated times.

### Live cell imaging

Cells were visualized at different time points after induction (6 h and 18 h) using an Olympus/Evident Spin IXplore SoRa microscope equipped with a 60x N.A. 1.5 oil objective (Nikon), a Spinning Disk (50 μm/50 µm SoRa) unit, and 488 nm Lasers (Coherent OBIS). For quantification, at least 100 cells were counted per condition and per experiment. For each condition, the number of cells displaying condensates were calculated relative to the total number of cells counted (with condensates or cytoplasmic distribution). At least three independent experiments with freshly transformed constructs were performed.

### Isopycnic sucrose density gradient

Expression of GAPR-1-GFP, [K7Q]GAPR-1-GFP and [K33Q]GAPR-1-GFP was induced for 18 h in SC selective medium supplemented with 2% galactose. Cells were mechanically lysed in R buffer (50 mM Tris-HCl, 1 mM EDTA, 50 mM DTT) containing fresh protease inhibitors (Aprotinin, Leupeptin, Pepstatin, PMSF) (Sigma-Aldrich, St. Louis, USA) with glass beads (0.25–0.5 mm, Carl Roth, Kalrsruhe, Germany). Cell lysates were centrifuged at 2000 x g for 10 min to remove unbroken cells and debris. Protein lysates (200 µg) were mixed with a sucrose stock solution to obtain a 40% (w/w) density in 1 mL volume, which was then overlayered with 1 ml of each 30%, 20%, and 10% sucrose buffers in SW60-2 tubes (Beckman Coulter, Brea, California, USA). After centrifugation at 60,000 rpm for 16 h, 12 fractions were collected from top to bottom of the gradients and were precipitated with chloroform/methanol (1:2). Samples were analyzed by SDS-PAGE and Western blotting for the presence of GAPR-1 using specific anti-GAPR-1 polyclonal antibodies.

### In-gel detection of N-myristoylation of GAPR-1

In-gel detection of *N*-myristoylation of proteins by metabolic labeling using an alkyne-tagged myristic acid analogue (13-tetradecynoic acid (Alk-14, MyBioSource/Bio-connect, Huissen, The Netherlands) has been described before [30–32] and applied here to yeast cells. To this end, yeast cells were pre-cultured in SC-2% raffinose medium overnight and then shifted to SC-2% galactose induction medium (starting from OD_600_ =0.1) at 30 °C for 18 h in the presence of 25 µM Alk-14. 25 OD_600_ cells were collected and resuspended in R buffer, supplemented with protease inhibitors (Aprotinin, Leupeptin, Pepstatin, PMSF). Subsequently, cells were mechanically lysed with glass beads (0.25–0.5 mm, Carl Roth, Kalrsruhe, Germany), and unbroken cells and cell debris were removed after centrifugation at 2000 x g for 10 min. Protein concentration was determined by Bradford Assay (Pierce™ Bradford Plus Protein Assay Reagent, Thermo Scientific™), and 500 µg total protein was collected for isolation of the membrane fraction by centrifugation at 17,000 x g for 30 min. The pellet fraction was washed by NT-50 buffer and subjected to centrifugation again. To solubilize the membrane fraction pellet, we first used 1/10th volume 2% SDS NT-50 buffer, then added 9/10th volume of NT-50 buffer to achieve a final concentration of 0.2% SDS in NT-50 [31].

For in-gel fluorescence analysis, 40 µg protein from cell lysates (1 mg/mL) was subjected to copper-catalyzed azide–alkyne cycloaddition (CuAAC). A freshly prepared click reaction mix was assembled containing azide–fluor 448 (10 mM), CuSO₄ (50 mM), tris(2-carboxyethyl)phosphine (TCEP; 50 mM, pH 7.5), and TBTA (10 mM) at a volumetric ratio of 1:2:2:1, respectively. The click mix was added to lysates at a final volume corresponding to 60 µL per 1 mL sample and incubated for 1 h at room temperature with shaking in the dark. The reaction was quenched by addition of EDTA (10 mM final concentration, pH 8).

SDS–PAGE and in-gel fluorescence detection: Proteins were separated by SDS–PAGE using 12% Criterion™ XT Bis–Tris gels (12 + 2 well, 45 µL; Bio-Rad, #3450117) run in XT MES running buffer (Bio-Rad, #1610789) according to the manufacturer’s instructions. Samples were mixed with 4× XT Sample Buffer (Bio-Rad #1610791) and loaded together with a PageRuler™ Plus Prestained Protein Ladder (10–250 kDa; Thermo Fisher Scientific, #26619). Electrophoresis was performed at 120 V for 10 min, followed by 200 V for approximately 30 min. Following electrophoresis, gels were briefly rinsed in deionized water and scanned for in-gel fluorescence using an Amersham Imager 600 operated in blue fluorescence mode (excitation ~ 460 nm). For downstream immunodetection, gels were subsequently processed for Western blotting using anti-GAPR-1 polyclonal antibodies.

### Acyl-biotin exchange

Lysis: Cell pellets were lysed in 100 µL full lysis buffer (50 mM triethanolamine (TEA), pH 7.5, 10 mM NaCl, 2 mM MgCl₂, 0.5% (v/v) NP-40, 0.2% (w/v) SDS, 1× EDTA-free protease inhibitor cocktail, 62.5 U/mL Benzonase, and 500 nM palmostatin B) and incubated for 10 min at RT with shaking at 800 rpm. Lysates were sequentially diluted with one volume of 50 mM TEA, pH 7.5, 10 mM EDTA, 6% (w/v) SDS and three volumes of 50 mM TEA, pH 7.5, 10 mM EDTA, 3% (w/v) SDS. Protein concentrations were determined using a bicinchoninic acid (BCA) assay (Thermo Scientific, 23227). Samples were adjusted to a final concentration of 1 mg/mL, and 1 mg total protein was used for all samples.

Reduction and alkylation: Samples were reduced with tris(2-carboxyethyl)phosphine (TCEP; 25 mM) for 30 min at room temperature with shaking at 800 rpm, followed by alkylation with N-ethylmaleimide (NEM; 15 mM) for 1 h under the same conditions. Samples were then adjusted to 4 M urea, supplemented with NEM to a final concentration of 30 mM, and incubated for 3 h at room temperature with shaking. Proteins were precipitated by methanol/chloroform precipitation and resuspended in dissolving buffer (50 mM TEA, pH 7.5, 150 mM NaCl, 5 mM EDTA, 2% (w/v) SDS). A second reduction (12.5 mM TCEP, 30 min) and alkylation (25 mM NEM, 1 h) was performed at room temperature with shaking. Proteins were precipitated by four consecutive methanol/chloroform precipitations and resuspended in dissolving buffer for subsequent steps.

Thioester biotinylation and enrichment: Samples were divided into two equal aliquots and incubated with HPDP-biotin (1.67 mM, in DMF/Milli-Q water, 2:1; Cayman Chemical, 16459-50) for 5 min, 25 °C with shaking at 800 rpm shaking. One aliquot was treated with 1 M hydroxylamine (HA), pH 7.5, for 2 h, RT with shaking at 800 rpm, while the corresponding control aliquot was treated with Milli-Q under identical conditions. Proteins were precipitated by three consecutive methanol/chloroform precipitations, resuspended in 50 mM TEA, pH 7.5, 150 mM NaCl, 5 mM EDTA, 2% (w/v) SDS, and diluted to a final concentration of 0.1% SDS using 50 mM TEA, pH 7.5, 150 mM NaCl, 5 mM EDTA.

Samples were incubated with 50 µL NeutrAvidin agarose beads, pre-washed with 50 mM TEA, pH 7.5, 150 mM NaCl, 5 mM EDTA, 0.1% (w/v) SDS, for 2 h at RT with end-over-end rotation. Beads were washed three times with 50 mM TEA, pH 7.5, 150 mM NaCl, 5 mM EDTA, 0.1% SDS and four times with 50 mM TEA, pH 7.5, 150 mM NaCl, 5 mM EDTA. Bound proteins were eluted by incubation with 50 mM TCEP, 50 mM TEA, pH 7.5, 5 mM EDTA, 0.1% (w/v) RapiGest prepared in HPLC-grade water for 30 min at room temperature with end-over-end rotation. Eluates were neutralized by addition of 50 mM HEPES, pH 8, and transferred to fresh tubes for downstream analysis.

### Protein expression and purification

Expression and purification of GAPR-1 (wtGAPR-1; UniProtKD ID: Q9H4G4 and mutant GAPR-1) have been described before [5, 33]. Briefly, the proteins were overexpressed in *Escherichia coli* XL-1 Blue host cells and induced by 0.5 mM IPTG overnight at 18 °C. The plasmids carrying wtGAPR-1 and mutants are listed in Table 2. After cell pelleting and homogenization with a high pressure homogenizer (Avestin, Mannheim, Germany), soluble proteins were collected by a two-step centrifugation for 30 min at 14,000 x g, followed by 30 min at 100,000 x g. GAPR-1 was purified by cation exchange chromatography using SP Sepharose FF (GE Healthcare, U.S.A.) and with a linear gradient of 0–400 mM NaCl in 25 mM Tris, pH 8.0. Protein buffer was exchanged to NT-50 buffer (25 mM Tris, 50 mM NaCl, pH 7.4).

For the expression and purification of GAPR-1-GFP and mutant ([K7Q]GAPR-1-GFP), a C-terminal 6xHis tag was added after the GAPR-1-GFP sequence. Expression in *E.coli* was performed as described above. Protein purification was conducted using ProBond™ Nickel-Chelating Resin, according to the manufacturer’s instructions. Briefly, bacterial cells were resuspended in Native Binding Buffer (50 mM NaH_2_PO_4_, 500 mM NaCl, pH 8.0) supplemented with protease inhibitors (Aprotinin, Leupeptin, Pepstatin, PMSF). Protein bound to the column was washed and subsequently eluted with Imidazole Buffer (50 mM NaH_2_PO_4_, 500 mM NaCl, 250 mM Imidazole, pH 8.0). The eluted protein was concentrated, and buffer was exchanged to NT-50 using Amicon^®^ Ultra 15 ml filters. The purity of the isolated proteins was confirmed by SDS-PAGE followed by Coomassie Blue staining and western blotting. All proteins were snap-frozen in liquid nitrogen and stored in aliquots at −80 °C until further use.

### Dot-blot assay

PIP strip membranes (Echelon Biosciences Inc. P-6001) were blocked with 3% BSA in PBS-T buffer overnight at 4 °C. Subsequently, the membranes were incubated with the protein of interest (0.5 µg/ml) in PBS-T supplemented with 3% BSA for 1 h at RT with gentle agitation. The membranes were washed three times by PBS-T for five to ten minutes between each step. Subsequently, the membranes were incubated for 1 h with a rabbit polyclonal anti-GAPR-1 antibodies at RT. A peroxidase-conjugated goat anti-rabbit antibody (Thermo Fisher Scientific, Massachusetts, USA) was used as secondary antibody. Western blot analysis was performed using the SuperSignal™ reagents (Thermo Scientific, Massachusetts, USA) and ChemiDoc™ MP Imaging system (BioRad, Hercules, California, USA).

### Thioflavin T staining fluorescence assay

Kinetics of zinc-induced GAPR-1 amyloid-like aggregation in the presence of heparin was monitored by Thioflavin T (ThT) fluorescence as described before [10, 11]. Briefly, reaction mixtures contained 15 µM GAPR-1, 37.5 µM heparin, 100 µM Zn^2+^ and 50 µM ThT dye (ThT, Sigma-Aldrich, USA) in NT-50 buffer. These mixtures were incubated in sealed 96-well plates (Flat Clear Bottom Black Polystyrene, Corning, USA.) at 37 °C for 24 h. Fluorescence was measured in a CLARIOstar microplate reader (BMG Labtech, Germany). ThT fluorescence emission was recorded at 488 nm after excitation at 449 nm, with agitation before every measurement.

### Dynamic light scattering (DLS)

Isothermal Dynamic Light Scattering (10 acquisitions, 5 s each) was used for particle sizing. 15 µM GAPR-1 was incubated with 37.5 µM heparin and 100 µM Zn^2+^ at RT. After incubation, samples were immediately loaded into High Sensitivity glass capillaries (10 µl each, *n* = 3). Capillaries were sealed with paste, following manufacturer’s instructions (NanoTemper Technologies GmbH, Munich, Germany). Measurements were started at 25 °C and subsequently heated up to 37 °C for incubations. Triplicates of ten consecutive DLS measurements of each capillary were performed in a Prometheus Panta at 100% laser power (NanoTemper Technologies GmbH, Munich, Germany). The hydrodynamic radius of the samples was determined with the associated software.

### Lentiviral production and transduction

Lentiviruses were produced using a 3rd generation packaging system (pRRLsin transfer vector with GAPR-1 inserts, pMDLg/pRRE, pRSV-Rev, pVSV-G). HEK293 cells in 100 mm dishes (70% confluency) were transfected with PEI (55 µg/plate) and plasmids (0.75 pmol/plate each pVSV-G, pRSV-Rev; 1.0 pmol/plate pMDLg/pRRE; 1.0 pmol/plate transfer vector; amounts per standardized spreadsheet). Supernatant was harvested 48 h post-transfection, aliquoted, and stored at − 80 °C.

Wild-type HeLa CCL-2 (ATCC) cells in 6-well plates (40% confluency) were transduced with 0.5 ml lentiviral supernatant + 0.5 ml 2× transduction enhancer (20 µg/ml DEAE-dextran) for 6 h, then medium refreshed. Puromycin selection started 48 h post-transduction (2 µg/ml); kill curve (0.1–2 µg/ml) determined in 96-well plates by CellSens imaging (Olympus CKX53). Stable lines maintained in T-75 flasks with 1 µg/ml puromycin.

### HeLa cell immunofluorescence

HeLa cells on glass coverslips were cultured in DMEM GlutaMAX (Gibco) + 10% Fetal Bovine Serum (FBS) + 1 µg/ml doxycycline hyclate (Sigma-Aldrich) to induce GAPR-1 for 24 h. Autophagy was induced with 20 µM Tat-beclin 1 [6] in acidified OPTI-MEM (0.15% 6 M HCl) for 1 h at 37 °C. Cells were fixed with 3.5% PFA/PBS (30 min, RT), incubated with anti-SQSTM1/p62 primary antibody (Abnova) (1 h, RT), then fluorophore-conjugated secondary antibody ((Donkey Anti-mouse Alexa Fluor^®^ 568, Abcam) (1 h, RT, dark) and finally nuclei staining with DAPI (20 min, dark). Confocal imaging used Nikon A1R/STORM (consistent settings); images processed in FIJI/ImageJ.

### Liposomes and samples preparation for Imaging

Liver PI, POPC, Liss Rhod-PE Lipids (Avanti Polar Lipids) were dissolved in chloroform/methanol (1:2) and stored at −20 °C. Lipids were mixed at desired molar ratios (100% POPC with 0.2% Liss Rhod-PE; 10%PI: 90% POPC + 10%PI + 0.2% Liss Rhod-PE; 20%PI: 80% POPC + 20%PI + 0.2% Liss Rhod-PE) in Eppendorf tubes, dried under a nitrogen gas stream, and followed by a 1 h vacuum treatment to remove any residual organic solvent. The lipid films were stored at −20 °C before use. To prepare liposomes, the lipid films were resuspended in NT-50 buffer to a final concentration of 6.8 mM. Samples were vortexed 3–4 times for 30 s each. The liposome mixtures were sonicated for 30 s on ice at medium level with three to five repetitions until the solution turns clear (from milky), followed by 1 h 37 °C incubation [8, 34].

All protein aliquots were centrifuged at 100,000 x g for 30 min at 4 °C prior to the experiments. 2 µM GAPR-1-GFP was mixed with 200 µM 0.2% Liss Rhod-PE labeled liposomes in NT-50 buffer or in 200 mM NaCl for the desired time at 37 °C.

Samples were analyzed using an Olympus/Evident Spin Ixplore SoRa microscope with a 60x oil lens. Samples (10 µL) were spotted on a 10-well chamber slide. Bright field and fluorescent images were captured at 20 °C.

### Size analyses of GAPR-1 assemblies

All images of GAPR-1-GFP incubated with liposomes were captured under identical conditions (the laser power, detector gain, resolution, scanning speed, etc.). At least 10 independent images were recorded for each experimental group. Image analysis was performed with ImageJ software. Fluorescence dots with sizes ranging from 0 to 150 pixels (equivalent to 1.75 µm^2^) were selected for analysis.

### Western blot analysis of GAPR-1 oligomerization

GAPR-1 oligomerization was analyzed by Western blot analysis. 5 µM GAPR-1 protein was incubated with 1 mM 100% POPC or 1 mM 20% PI liposomes in 25 mM Tris-HCl, 0.1 mM EDTA, 150 mM NaCl, pH 7.4 at 37 °C. From each incubation, samples were collected after 90 min, 1 day, and 3 days of incubation.

For yeast cells: 10 OD units of cells were collected by centrifugation, washed with sterile water and lysed with 200 µl of Y-PER™ reagent (Thermo Scientific, Waltham, MA, USA) containing fresh protease inhibitors (Aprotinin, Leupeptin, Pepstatin, PMSF) (Sigma Aldrich, St. Louis, MO, USA). Lysed cells were incubated for 20 min at room temperature, followed by a 15 min centrifugation at 13,000 rpm.

For stable HeLa cell lines: Cells were washed with PBS, scraped, resuspended in homogenization buffer (25 mM Tris, 50 mM NaCl, 0.25 M sucrose, 3mM imidazole pH 7.4) containing fresh protease inhibitors and homogenized by syringe passage through 26G needle. Post-nuclear supernatant (PNS) was obtained by centrifugation at 1,500 × g for 5 min. PNS was then centrifuged at 100,000 × g for 1 h to separate membrane (pellet) and cytosolic (supernatant) fractions. Membrane pellets were resuspended in the same volume of NT-50 buffer (25 mM Tris, 50 mM NaCl, pH 7.4) as the supernatant (cytosol).

Protein samples (20 µg or as indicated for each experiment) were mixed with Laemmli sample buffer, boiled for 5 min, and separated by SDS/PAGE. The separated proteins were then transferred onto 0.45 μm Nitrocellulose membranes (Amersham™ Protran^®^ GE Healthcare) by Western blotting at 100 V for 60 min. GAPR-1 protein was visualized with a rabbit polyclonal anti-GAPR-1, caveolin with a rabbit poly clonal anti-caveolin (BD Transduction Laboratories) antibody and HRP-labeled goat anti-rabbit secondary antibodies as described above for the dot-blot assay.

### Statistical analysis

Data were analyzed using GraphPad Prism 9 (San Diego, CA, USA) Software and are presented as mean ± SD of at least three independent experiments. The significance of differences was calculated using a two-tailed unpaired T-test unless indicated. P values less than 0.05 were considered to indicate a significant difference.

## Results

### GAPR-1 membrane orientation and lipid binding sites

**Fig. 1:**
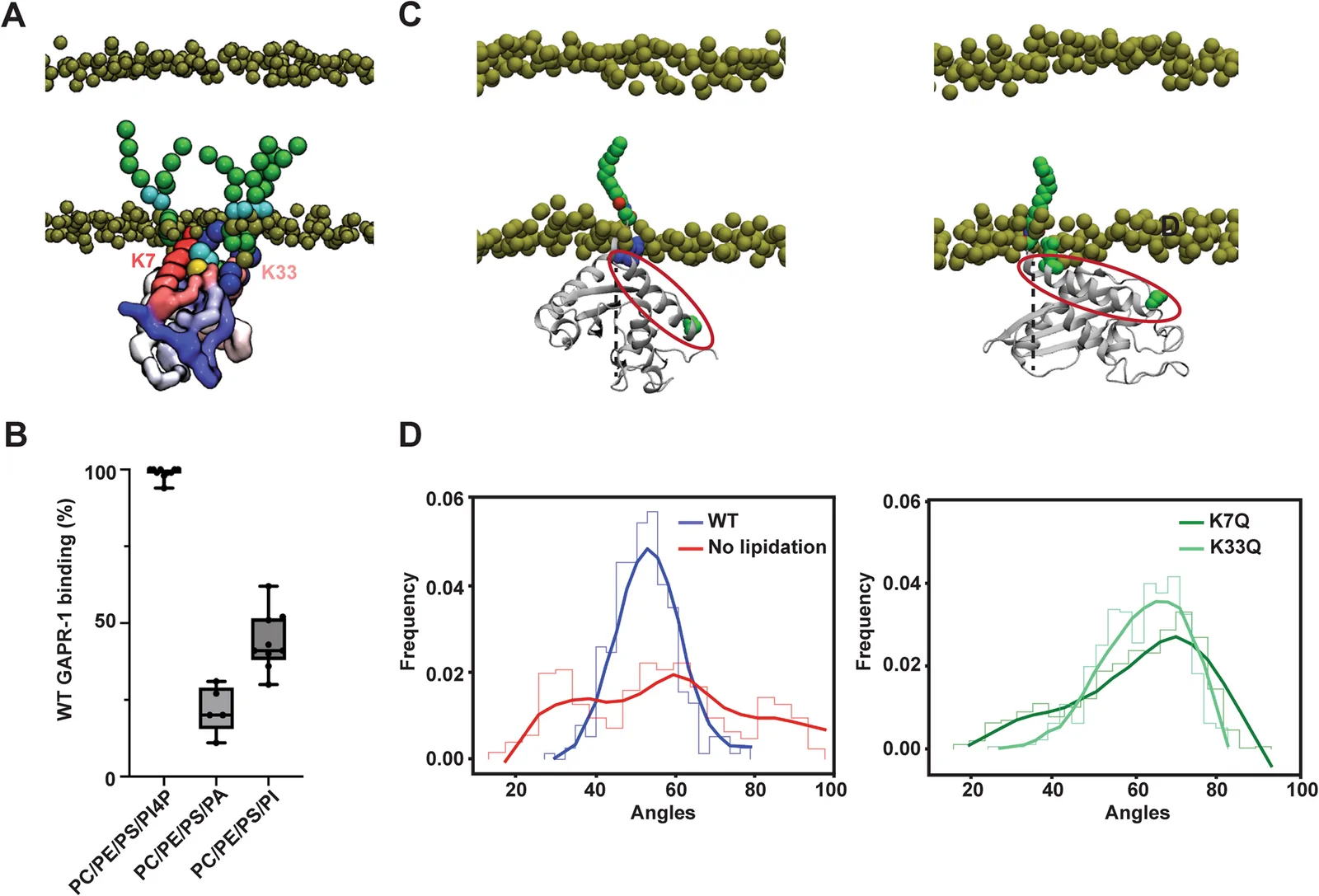
GAPR-1 membrane orientation and lipid binding sites **A**. CG-MD simulations of wtGAPR-1 interactions with negatively charged lipid bilayers composed of PC/PE/PS/PI4P reveal a dominant pose for GAPR-1 binding to the membrane. GAPR-1 is colored red> blue according to residue number. Two key lysine residues are shown. Membrane lipid phosphate beads are shown as gold spheres. Two bound PI4P lipids are shown in green spheres/cyan spheres; **B**. CG-MD simulations of wtGAPR-1 binding to lipid membranes as described in Panel **A**, but with varying negatively charged lipids as indicated in the legend (1. PC/PE/PS/PI4P, 2. PC/PE/PS/PA, 3. PC/PE/PS/PI); **C**. AT-MD simulations of myristoylated GAPR-1 (left image) and non- myristoylated GAPR-1 (right image) on lipid membranes, converted from the pose shown in Panel **A;** **D**. Left panel: The orientation of membrane-docked myristoylated and non-myristoylated GAPR-1 was quantified by analysing the frequency of wtGAPR-1 structure distributions in various coordinates, using the angle between the N-terminal helix (indicated by red ovals in panel **C**) and the vertical axis (relative to the plane of the membrane, indicated by dotted line). For each condition, 3 × 0.65 µs of simulation was analyzed. Plots show the data binned into histograms, shown as a thin line, with the center of each bin connected with a thick line; Right panel: quantification of the orientation of membrane-docked myristoylated [K7Q]GAPR-1 and myristoylated [K33Q]GAPR-1 on lipid membranes

Our previous studies revealed that membranes contribute to GAPR-1 amyloid-like oligomerization in vitro and condensate formation in vivo. To investigate the molecular mechanism underlying this phenomenon, we performed coarse-grained (CG) and atomistic (AT) molecular dynamics (MD) simulations of GAPR-1. Initially, we used CG-MD to assess how soluble GAPR-1 binds to biological membranes. These analyses provide information both on the likelihood of the interaction, but also on specific interactions made between GAPR-1 and lipids in the membrane [35]. For this, we initially used a membrane comprised of phosphatidylcholine: phosphatidylethanolamine: phosphatidylserine: phosphatidylinositol-4-phosphate (PC: PE: PS: PI4P) at a molar ratio of 50:30:10:10. Non-myristoylated GAPR-1 was positioned approximately 8 nm from the membrane in 10 independent starting orientations, and for each system 15 µs simulations were run. These data reveal that GAPR-1 has a high affinity for the membrane, with the protein spending ca. 96% of the simulation time from 2 to 10 µs bound to the membrane. Most of this interaction time is dominated by a single dominant pose, driven by the amino acids Lys7 and Lys33. This results in a binding orientation with a tilted N-terminus (Fig. 1A).

We previously showed that non-myristoylated wtGAPR-1 binds to liposomes containing negatively charged lipids such as PS, phosphatidylglycerol (PG), phosphatidic acid (PA), and phosphatidylinositol (PI) [34]. Similarly, non-myristoylated wtGAPR-1 binds a variety of negatively charged lipids that were spotted on nitrocellulose (Suppl. Figure 1 A). To probe this further, we repeated the analyses in Fig. 1A for a range of different membrane compositions. The data reveal a stronger preference for PI4P than for either PI or PA (Fig. 1B). PS (present in all conditions) did not associate with GAPR-1, unlike PI4P for which an average of 4 lipids could bind to GAPR-1 at once (Suppl. Figure 1B). The binding of multiple PI4P lipid molecules suggest a broad binding area for lipids with some selectivity for specific negatively charged lipids (PI4P˃PI˃PA˃PS).

To understand the dynamics and interaction of wtGAPR-1 when bound in this position, we converted a post CG-MD snapshot to a higher resolution atomistic description and ran additional simulations. These were run either with or without a myristate tail added to Gly2 of wtGAPR-1. To quantify the dynamics of wtGAPR-1 at the membrane, we computed the angle between the N-terminal α-helix (Fig. 1C, red oval) and the vertical z-axis, relative to the membrane (Fig. 1C, dotted line), for each frame of the simulation. wtGAPR-1 maintained a relatively stable orientation at the membrane, as indicated by the narrow distribution angle range with maximal frequency at approximately 55 degrees (Fig. 1D, left panel). When myristate was not added to GAPR-1, whilst GAPR-1 remained docked on the membrane due to ionic interactions, the preferred GAPR-1 orientation was lost, as can be seen by a much wider range of orientations samples (Fig. 1D, left panel). These results indicate that myristate plays an important role in the orienting GAPR-1 in a specific angle at the membrane.

The contribution of Lys7 and Lys33 to the tilted position of the N-terminus of membrane-docked GAPR-1 was investigated by mutating Lys7 or Lys33 to glutamine ([K7Q]GAPR-1 and [K33Q]GAPR-1). Comparison of the frequency of structure distributions of these mutated GAPR-1 proteins with wtGAPR-1 showed that [K7Q]GAPR-1 and [K33Q]GAPR-1 shifted the preferential orientation to approximately 70 and 68 degrees, respectively (Fig. 1D, right panel).

**Fig. 2:**
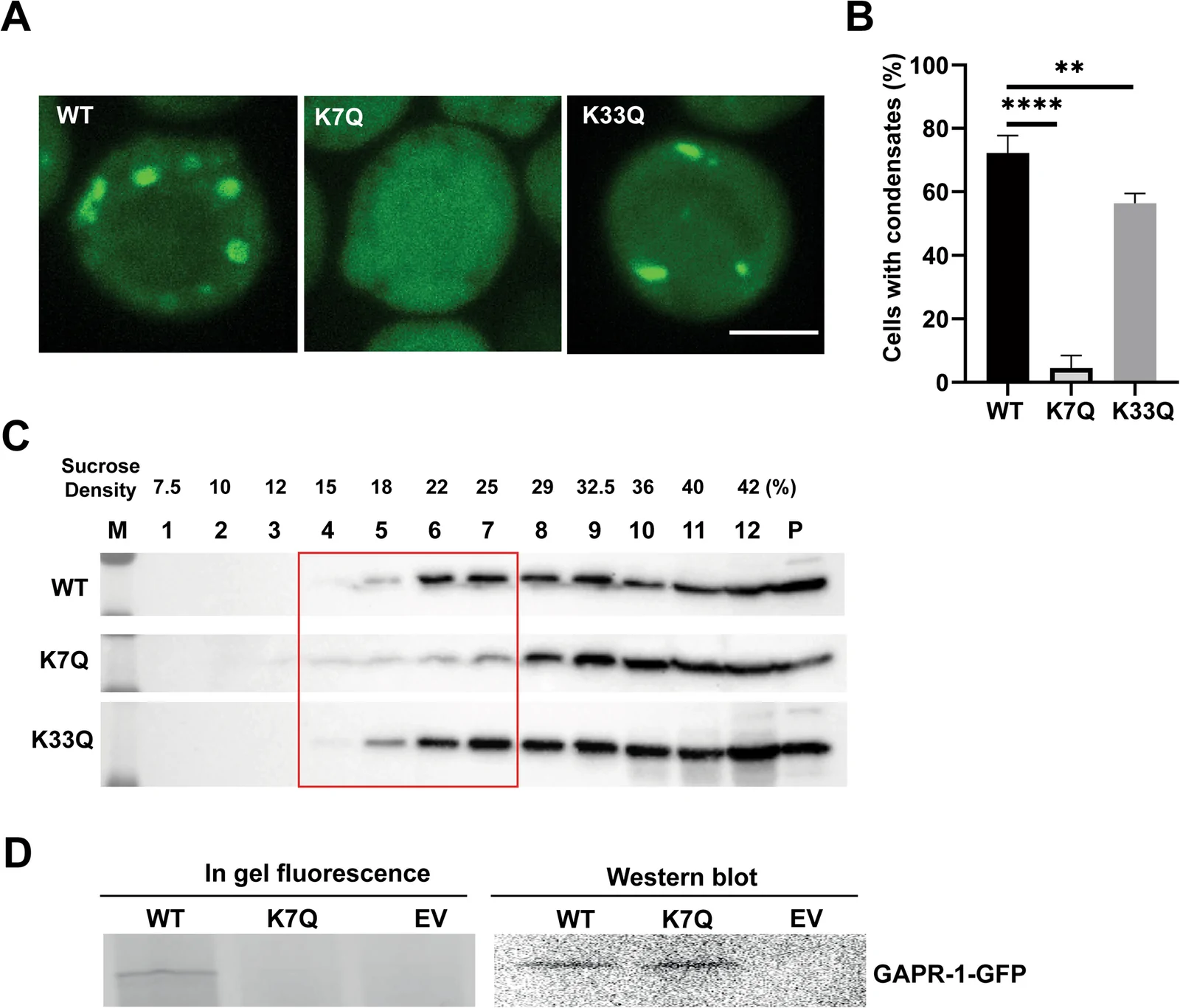
Role of Lys7 in GAPR-1 oligomerization and membrane association **A**. Representative fluorescence microscopy images of yeast cells after 18 h expression of wtGAPR-1-GFP, [K7Q]GAPR-1-GFP or [K33Q]GAPR-1-GFP; **B**. Quantification of cells displaying GAPR-1-GFP condensates. Significance of differences was calculated with two-tailed unpaired t-test (ns ≥ 0.05, *p < 0.05, **p < 0.01, ***p < 0.001, ****p < 0.0001); **C**. After 18 h expression of wtGAPR-1-GFP, [K7Q]GAPR-1-GFP or [K33Q]GAPR-1-GFP, yeast cells were collected and homogenized. Cell lysates were subjected to sucrose density centrifugation at 60,000 rpm for 16 h. 12 fractions and the pellet (P) were collected and analyzed for the presence of GAPR-1 by SDS-PAGE and Western blot using anti-GAPR-1 antibodies. The red box indicates the light membrane fraction; **D**. In-gel fluorescence detection of *N*-myristoylation of GAPR-1 WT and [K7Q]GAPR-1. Yeast cells were transformed with wtGAPR-1, [K7Q]GAPR-1 or with an empty vector (EV) and proteins were expressed for 18 h in yeast cells and with the lipid probe Alk-14. After cell lysis, proteins in the isolated membrane fraction were analysed for the presence of Alk-14 by the copper (I) catalyzed azide-alkyne cycloaddition (CuAAC) with Azide-Fluor 545 directly after SDS-PAGE (right). A fluorescent signal corresponding to GAPR-1–GFP was detected exclusively in the WT sample, whereas no signal was observed in the K7Q mutant or EV control. Western blot analysis confirmed the presence of GAPR-1–GFP in both WT and K7Q samples, western blot was performed on a gel ran with identical conditions as for the in-gel fluorescence

As Lys7 and Lys33 affect the interaction of GAPR-1 with membranes, we tested whether these lysines are also important for GAPR-1 condensate formation. To this end, we mutated these two amino acids individually and evaluated their influence on GAPR-1 condensate formation in *Saccharomyces cerevisiae*. After overexpression of GFP-tagged GAPR-1 constructs in yeast cells for 18 h, we compared the wtGAPR-1-GFP phenotype to the [K7Q]GAPR-1-GFP and [K33Q]GAPR-1-GFP phenotypes using live-cell fluorescence microscopy (Fig. 2A). Whereas 72% of the yeast cells contained condensates after 18 h expression of wtGAPR-1-GFP, only 4.6% of the yeast cells contained condensates after expression of [K7Q]GAPR-1-GFP, despite similar expression levels (Suppl. Figure 2). [K33Q]GAPR-1-GFP showed a modest decrease in the number of cells forming condensates to 56% (Fig. 2A and B).

GAPR-1 oligomerization and condensate formation are catalyzed by interactions with membranes and therefore, these results could indicate that the K7Q mutation reduces the binding of GAPR-1 to membranes. To investigate this, we overexpressed wtGAPR-1-GFP, [K7Q]GAPR-1-GFP, and [K33Q]GAPR-1-GFP in yeast. After 18 h of overexpression, the cells were harvested, and the cell lysates were subjected to sucrose density centrifugation at 60,000 rpm for 16 h. 12 fractions and the pellet were collected and analyzed for the presence of GAPR-1 by SDS-PAGE and Western blot. As shown in Fig. 2C, [K33Q]GAPR-1-GFP behaved very similar to wtGAPR-1-GFP and floated up to 15% sucrose density. In contrast, [K7Q]GAPR-1-GFP showed less association with membranes and only small amounts of protein were observed in the light fractions (sucrose density ranging from 15% to 25%).

The reduced membrane affinity of [K7Q]GAPR-1-GFP is reminiscent of the reduced membrane binding of non-myristoylated wtGAPR-1 in yeast cells [16]. The consensus sequence for protein myristoylation [36, 37] indicates that a lysine at position 7 is not essential for protein myristoylation, but it may affect GAPR-1 myristoylation. Therefore, we determined whether the K7Q mutation affected GAPR-1 myristoylation during overexpression in yeast cells. We first metabolically labeled cultured yeast cells with a lipid probe (myristic acid alkyne (Alk-14)) for 18 h. During these incubations, the internalized Alk-14 is incorporated into substrate proteins by N-myristoyl-transferase. After cell lysis, the lipid probe-labeled proteins in the membrane fraction were analyzed after copper(I) catalyzed azide-alkyne cycloaddition (CuAAC) with Azide-Fluor 545, which enabled in-gel fluorescence visualization of incorporated lipid probes directly after SDS-PAGE. As shown in Fig. 2D, wtGAPR-1-GFP was myristoylated, whereas [K7Q]GAPR-1-GFP was also not myristoylated. The presence of similar amounts of GAPR-1 protein was confirmed by immunoblotting (Fig. 2D, right panel).

Near the N-terminus and close to Lys33 there is also a cysteine (Cys32) that could potentially be subject to palmitoylation. To determine whether wtGAPR-1 can be S-palmitoylated, we performed Acyl-biotin exchange (ABE)-labeling [38] on stably transfected Hela cells, expressing wtGAPR-1 or [K7Q]GAPR-1-GFP. In contrast to Caveolin 1, GAPR-1 does not become palmitoylated (Suppl. Figure 3 A). In addition, expression of [C32S] GAPR-1 in yeast cells did not affect the GAPR-1 condensate formation (Suppl. Figure 3B). These results show that palmitoylation does not contribute to the membrane binding or condensate forming characteristics of GAPR-1.

In summary, our results show that the GAPR-1 interaction with membranes is driven by myristoylation and two positively charged amino acids (Lys7 and Lys33), causing a specific and stable membrane orientation. The K7Q mutation in GAPR-1 not only directly affects the orientation of GAPR-1 at the membrane as observed in silico (Fig. 1D), but in vivo it may also indirectly affect the rotational motion of GAPR-1 association with lipid membranes due to the concomitant lack of myristoylation.

### Role of K7 in GAPR-1 binding to liposomes and protein aggregation

**Fig. 3:**
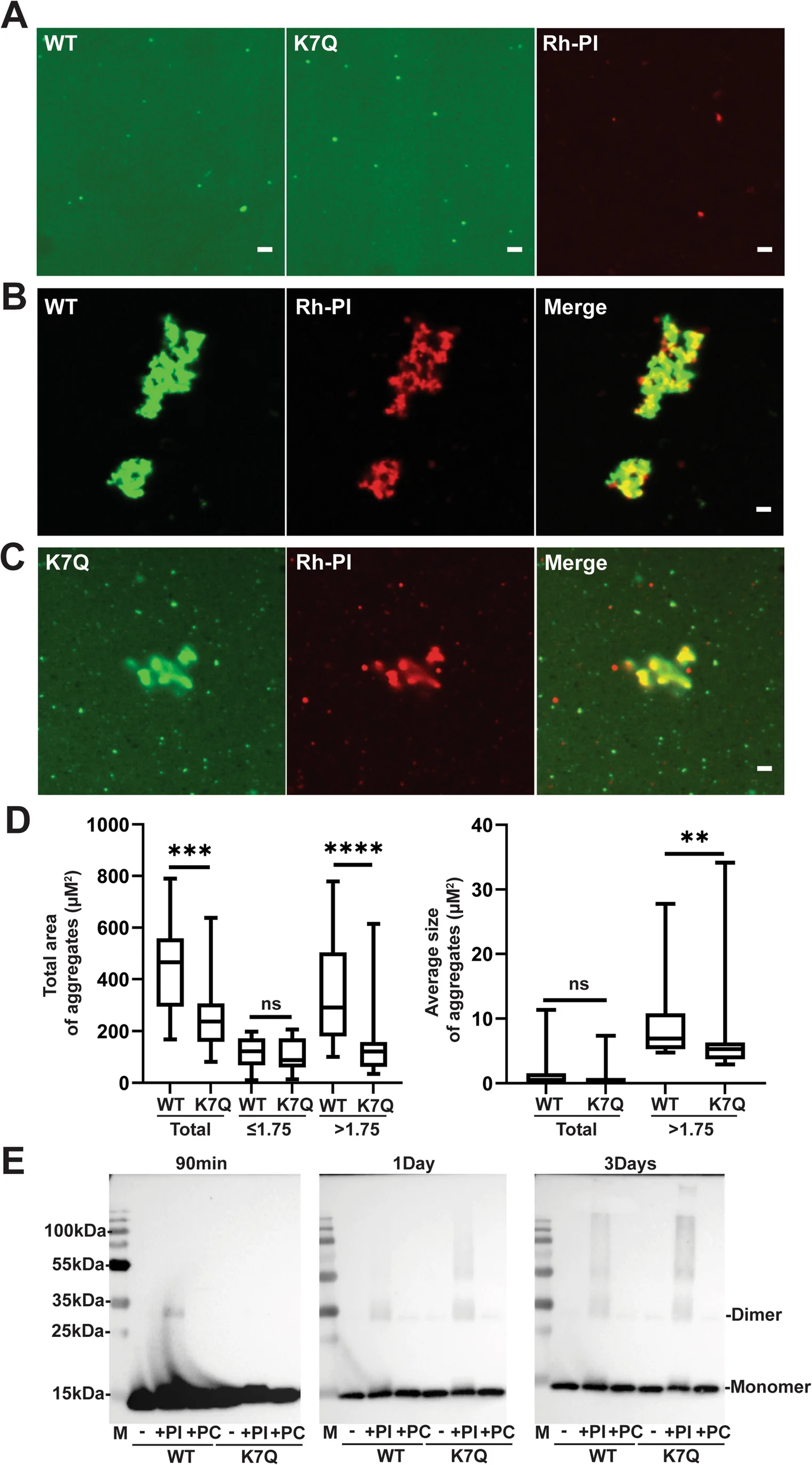
Role of K7 in GAPR-1 binding to liposomes and protein aggregation** A**. Representative fluorescence microscopy images after incubation of purified recombinant non-myristoylated wtGAPR-1-GFP (2 µM), [K7Q]GAPR-1-GFP (2 µM), or Rhodamine-PE labeled 10% PI liposomes (Rh-PI, 200 µM) for 1 day at 37 ℃; **B**. Representative fluorescence microscopy images after incubation of non-myristoylated wtGAPR-1-GFP (2 µM) with Rh-PI (200 µM) at 37 ℃ for 1 day; **C**. Representative fluorescence microscopy images after incubation of non-myristoylated [K7Q]GAPR-1-GFP (2µM) with Rh-PI (200 µM) at 37 ℃ for 1 day. Scale bars are 2 μm; **D**. Quantitative analysis of aggregate sizes formed by incubation of wtGAPR-1-GFP and [K7Q]GAPR-1-GFP with Rh-PI as described for panel **B** and **C**. The left panel summarizes the total area of GFP- fluorescence positive particles and describes the total area of all particles, the total area of particles with sizes equal to or less than 1.75 µM^2^, and the total area of particles larger than 1.75 µM^2^. The Right panel summarizes the average size of GFP- fluorescence positive particles and describes the average size of all (Total) particles and average size of particles larger than 1.75 µM^2^. Significance of differences was calculated with Mann–Whitney U test (* *p* < 0.05, ** *p* < 0.01 and *** *p* < 0.001, **** *p* < 0.0001); E. Western blot analysis of wtGAPR-1 and [K7Q]GAPR-1 (5 µM) after incubation with or without liposomes (1 mM) at 37 °C for 90 min (left panel), 1 day (middle panel) and 3 days (right panel), respectively. Liposomes consisted of PC or PC/PI (80/20%) as indicated in Materials and Methods

Given the stronger phenotype of the Lys7 mutation versus the Lys33 mutation (both in MD dynamics and condensate formation), we explored the role of the specific GAPR-1 membrane orientation in more detail using the Lys7 mutation. To discriminate between a direct effect of the K7Q mutation and an indirect effect due to lack of myristoylation, we first compared the amyloid-like aggregation properties of non-myristoylated wtGAPR-1 with non-myristoylated [K7Q]GAPR-1. To investigate a direct role of Lys7 on GAPR-1 liposome-induced aggregation, we compared the aggregation characteristics of purified recombinant wtGAPR-1-GFP and [K7Q]GAPR-1-GFP after binding to negatively charged Rhodamine-PE labeled liposomes. Recombinant GAPR-1 is not myristoylated and hence, any observed differences between wtGAPR-1-GFP and [K7Q]GAPR-1-GFP can be attributed to the K7Q mutation. The aggregation of GAPR-1 was determined by fluorescence microscopy (Fig. 3A-C). When wtGAPR-1-GFP or [K7Q]GAPR-1-GFP was incubated without liposomes for 24 h, no protein aggregates were observed (Fig. 3A). When wtGAPR-1-GFP (Fig. 3B) or [K7Q]GAPR-1-GFP (Fig. 3C) was incubated with negatively charged liposomes for 24 h, large protein aggregates were observed. A dot blot assay confirmed that the protein loading amounts of wtGAPR-1-GFP and [K7Q]GAPR-1-GFP were the same (Suppl. Figure 4 A). Aggregate formation was not observed in the absence of negative charge (PI) or in the presence of elevated salt concentrations (Sup. Figure 4B), in agreement with ionic interactions playing a crucial role in the interaction of wtGAPR-1-GFP and [K7Q]GAPR-1-GFP with liposomes. Aggregate formation was quantified using ImageJ. Consistent with microscopic observations, the total aggregate particle area formed by [K7Q]GAPR-1-GFP was significantly smaller than that formed by wtGAPR-1-GFP (Fig. 3D). When distinguishing between small (≤ 1.75 μm², representing background, cf. Figure 3A) and large (> 1.75 μm²) particles, a significant difference between wtGAPR-1-GFP and [K7Q]GAPR-1-GFP was observed for particles > 1.75 μm², both in terms of total particle area and average size (Fig. 3D). These results suggest that the oligomeric and/or amyloidogenic properties of GAPR-1 are affected by the K7Q point mutation.

To further investigate GAPR-1 oligomer formation after liposome binding, we performed Western blot analysis as previously described [9]. wtGAPR-1-GFP forms a dimer after 90 min of incubation with negatively charged liposomes, and high molecular weight oligomers were observed after 3 days of incubation (Fig. 3E). In contrast, [K7Q]GAPR-1-GFP did not form detectable amounts of dimers after 90 min of incubation with negatively charged liposomes, but high molecular weight oligomers were observed after 1 day of incubation (Fig. 3E). Under all conditions, the formation of GAPR-1 dimers and oligomers was specific for the presence of negatively charged lipids in the liposomes (Fig. 3E).

### Role of K7 in GAPR-1 oligomerization and amyloid-like aggregation

**Fig. 4:**
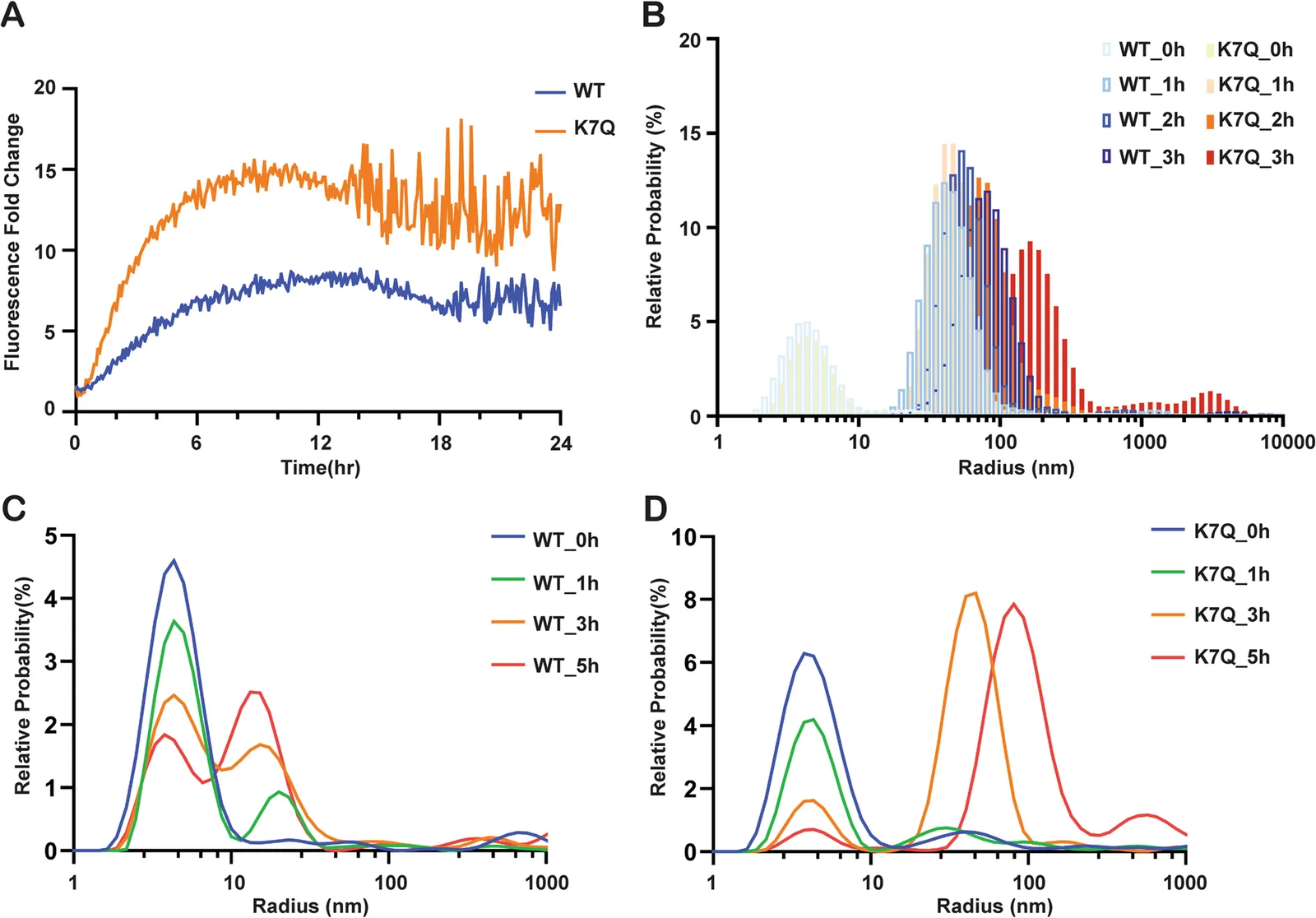
Role of K7 in GAPR-1 oligomerization and amyloid-like aggregation** A**. Kinetics of ThT fluorescence enhancement during incubation of 15 µM non-myristoylated purified recombinant wtGAPR-1 (WT, blue) or [K7Q]GAPR-1 (K7Q, orange) with 37.5 µM heparin and 100 µM Zn^2+^; **B**. Dynamic Light Scattering (DLS) analysis of wtGAPR-1 (blue) or [K7Q]GAPR-1 incubated with 37.5 µM heparin and 100 µM Zn^2+^ for 1, 2, 3 h at 37 ℃. Size distribution fits with the relative frequency of detected particle sizes (set to 100%) was plotted against the mean hydrodynamic radius from cumulant analysis by Prometheus Panta (NanoTemper Technologies, Germany); **C** and **D**. Dynamic Light Scattering (DLS) analysis of wtGAPR-1 (C) and [K7Q]GAPR-1 (D) incubated with 37.5 µM heparin for 0, 1, 3, 5 h at 37 ℃ in the absence of zinc ions. Size distribution fits with the relative frequency of detected particle sizes (set to 100%) was plotted against the mean hydrodynamic radius from cumulant analysis by Prometheus Panta (NanoTemper Technologies, Germany)

The effect of the K7Q mutation on the oligomeric and amyloidogenic properties was further analyzed in a recently developed in vitro assay system using negatively charged polysaccharide heparin [10]. Heparin is commonly used to study aggregation induction and kinetics in vitro of amyloidogenic proteins [10]. GAPR-1 forms amyloid-like aggregates in the presence of heparin and zinc ions that can be detected by Thioflavin T (ThT), a dye that specifically binds to β-sheet-rich fibrils. In this assay, purified recombinant [K7Q]GAPR-1 forms amyloid-like fibrils in the presence of Zn^2+^ and heparin with similar, if not improved efficiency as compared to wtGAPR-1 (Fig. 4A). We used dynamic light scattering (DLS) to monitor the formation of oligomeric intermediates upon incubation of GAPR-1 with heparin and Zn^2+^ at early stages (0–3 h). At the start of incubation (0 h), wtGAPR-1 and [K7Q]GAPR-1 are present in particles with a hydrodynamic radius of around 4 nm (Fig. 4B). During the 3 h incubation, [K7Q]GAPR-1 forms larger oligomeric structures as compared to wtGAPR-1, indicating that amyloidogenic oligomeric behavior is different from wtGAPR-1. This is further supported by incubation of GAPR-1 with heparin in the absence of metal ions (Fig. 4C and D). Under these conditions, wtGAPR-1 does not form amyloid-like aggregates [10], but during a 5 h incubation at 37 ֯C, the hydrodynamic radius of wtGAPR-1 oligomeric structures increases from 4 to ~ 12 nm (Fig. 4C). In contrast, the kinetics of [K7Q]GAPR-1 oligomer formation is faster and it forms larger oligomers. Already after 3 h of incubation, most of the [K7Q]GAPR-1 has formed oligomeric structures with a hydrodynamic radius of ~ 50 nm, which further increased to ~ 100 nm after 5 h of incubation. We excluded the possibility that K7Q interfered with the stability of the GAPR-1 protein using a thermal stability assay. The wtGAPR-1 and [K7Q]GAPR-1 stability appeared comparable under all conditions tested (in the presence or absence of heparin and/or Zn^2+^) (Suppl. Figure 5). Thus, differences in oligomeric behavior between wtGAPR-1 and [K7Q]GAPR-1 cannot be attributed to differences in protein stability. The combined results suggest that either the kinetics of oligomer formation or the type of oligomers formed by [K7Q]GAPR-1 are different from wtGAPR-1.

### [K7Q]GAPR-1 does not form homo-oligomers with wtGAPR-1

**Fig. 5:**
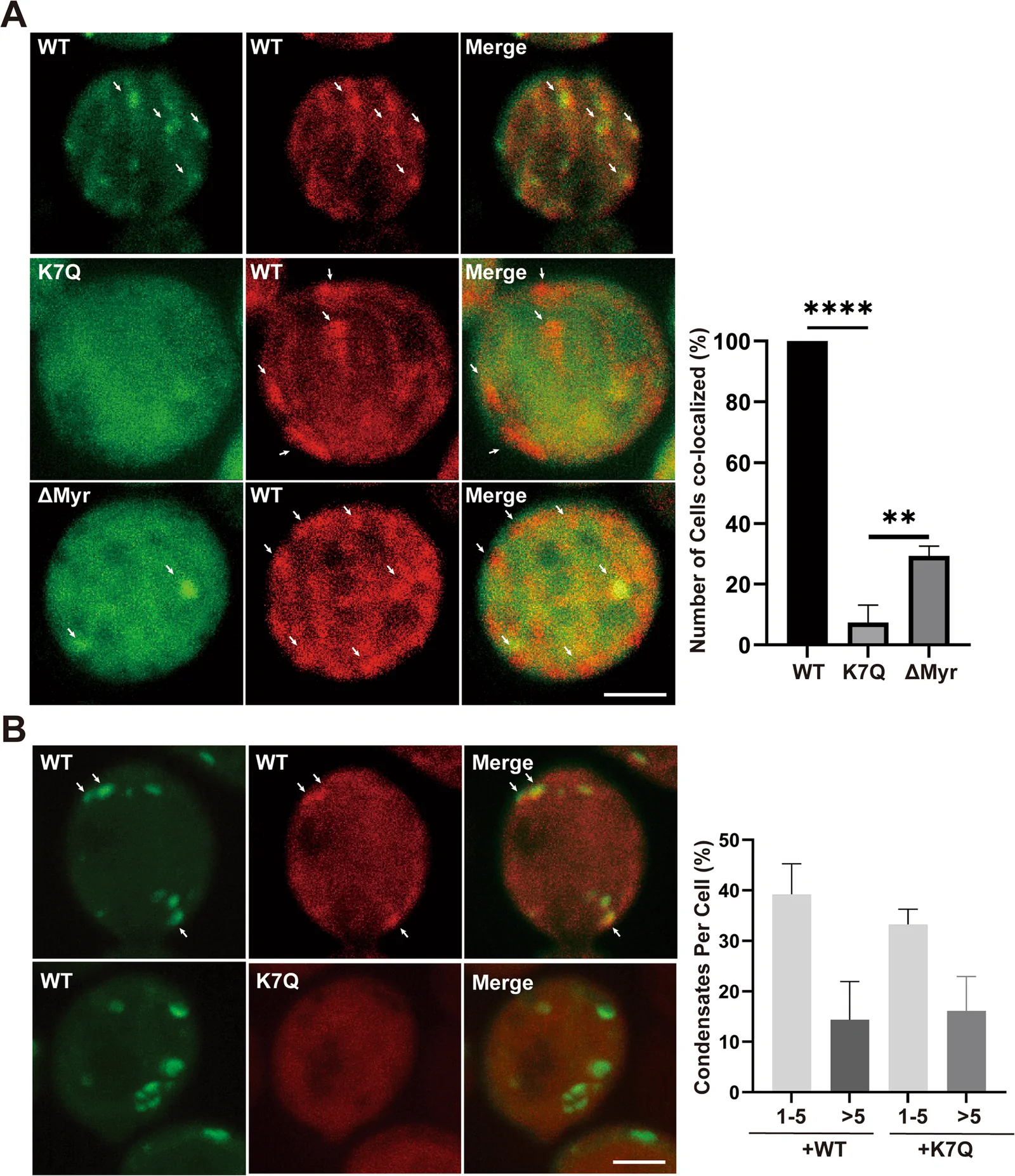
[K7Q]GAPR-1 does not form homo-oligomers with wtGAPR-1** A**. Representative live-cell fluorescence microscopy images of yeast cells after 6 h expression of constructs as indicated: Top panels show co-expression of wtGAPR-1-GFP and wtGAPR-1-mCherry; Middle panels show co-expression of [K7Q]GAPR-1-GFP and wtGAPR-1-mCherry; Bottom panels show co-expression [G2A]GAPR-1 (ΔMyr-GAPR-1-GFP) and wtGAPR-1-mCherry. Right panel shows quantitative analysis of cells displaying colocalization of wtGAPR-1-GFP, [K7Q]GAPR-1-GFP, and ΔMyr-GAPR-1-GFP with wtGAPR-1-mCherry. Significance of differences was calculated with two-tailed unpaired t-test (* *p* < 0.05, ** *p* < 0.01 and *** *p* < 0.001, **** *p* < 0.0001); **B**. Representative live-cell fluorescence microscopy images of yeast cells after 6 h co-expression of wtGAPR-1-GFP with wtGAPR-1-mCherry or [K7Q]GAPR-1-mCherry Colocalization was quantified by identifying wtGAPR-1-mCherry condensates first (red channel), then scoring K7Q-GFP overlap (green channel) in identical ROI. *n* > 100 cells/condition. Quantification of wtGAPR-1-GFP condensates (1–5 condensates per cell, more than 5 condensates per cell) after co-expression with wtGAPR-1-mCherry or [K7Q]GAPR-1-mCherry. Scale bar is 2 μm

To investigate possible differences between the types of oligomers formed by wtGAPR-1 and [K7Q]GAPR-1, we employed a recently developed novel screening assay that allows investigation of homotypic amyloidogenic protein-protein interactions [19]. In this assay system, co-expression of wtGAPR-1-mCherry and wtGAPR-1-GFP results in co-localization in condensates, suggesting that the differentially tagged proteins form homo-oligomers (Fig. 5A). In contrast, co-expression of wtGAPR-1-mCherry with [K7Q]GAPR-1-GFP did not result in significant co-localization of both proteins to the same condensates. After 6 h of co-expression, wtGAPR-1-mCherry was found in condensates, whereas [K7Q]GAPR-1-GFP remained largely in the cytosol. Quantification showed that of all wtGAPR-1-mCherry positive condensates (set at 100%), only 7.4% showed co-localization with [K7Q]GAPR-1-GFP condensates (Fig. 5A). Also, the number of wtGAPR-1-mCherry condensates per cell is not significantly affected by co-expression with [K7Q]GAPR-1-GFP (Fig. 5B). To exclude the possibility that the lack of co-localization to the same condensates is due to a lack of myristoylation of [K7Q]GAPR-1-GFP, we also compared the co-localization of wtGAPR-1-mCherry with [G2A]GAPR-1-GFP (ΔMyr-GAPR-1-GFP, which lacks a myristate group [16]). Upon co-expression with wtGAPR-1-mCherry, [G2A]GAPR-1-GFP is found in condensates in 29% of the wtGAPR-1 condensates (Fig. 5A). Thus, the combined results suggest that wtGAPR-1 and [K7Q]GAPR-1 do not interact to form homo-oligomers upon co-expression in yeast cells. This lack of interaction is not due to the absence of myristoylation in [K7Q]GAPR-1, although it may contribute to the severity of the phenotype. Instead, the phenotype likely reflects a different and incompatible membrane orientation of the K7Q mutant as compared to wtGAPR-1.

### [K7Q]GAPR-1 does not interact with beclin 1

**Fig. 6:**
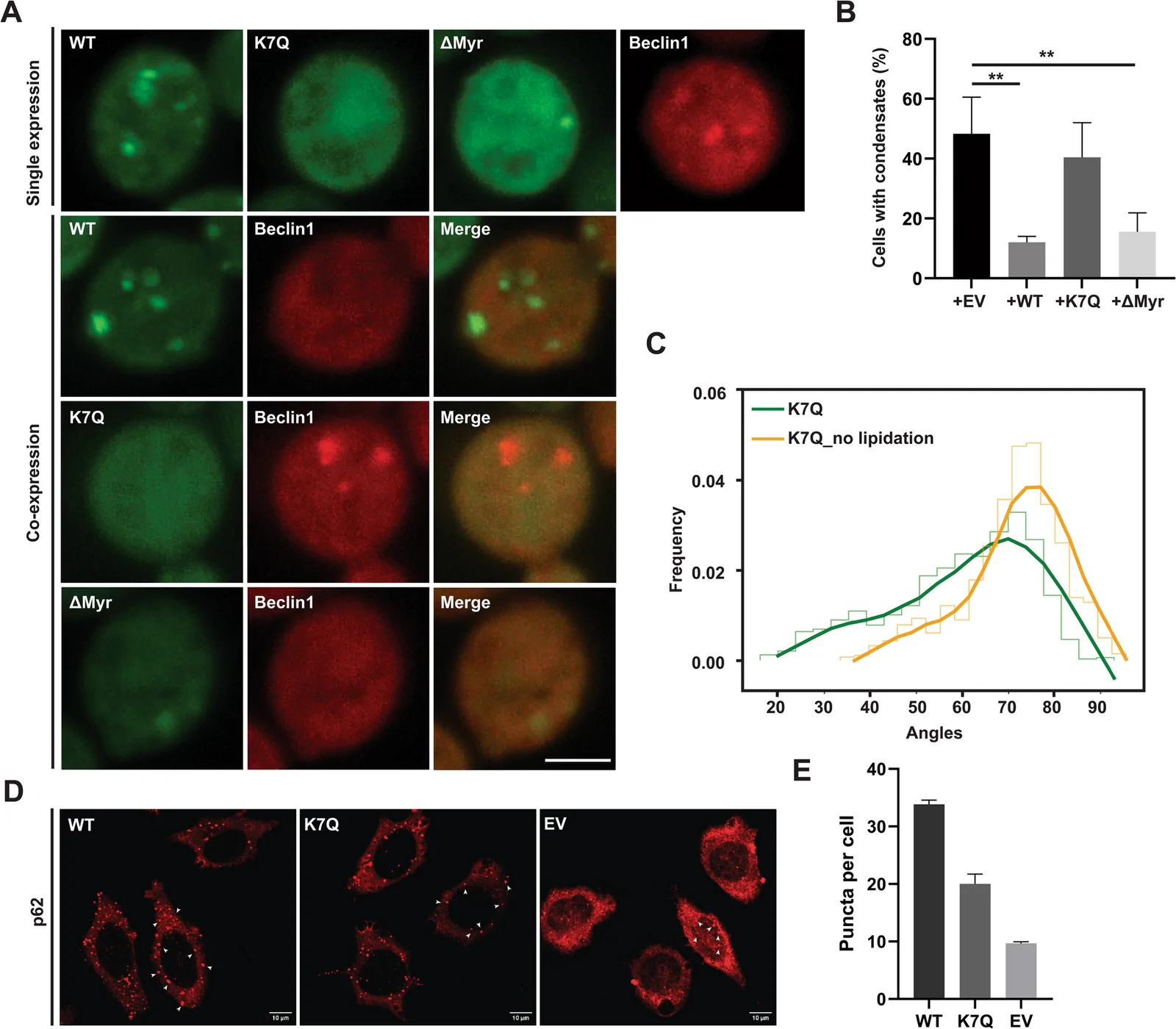
[K7Q]GAPR-1 does not interact with beclin 1 **A**. Representative live cell fluorescence microscopy images of yeast cells after 18 h of single expression or co-expression of wtGAPR-1-GFP, [K7Q]GAPR-1-GFP, ΔMyr-GAPR-1-GFP or beclin 1-mCherry as indicated. Scale bar is 2 μm;** B**. Quantification of yeast cells displaying beclin 1 condensates upon single expression or co-expression with wtGAPR-1-GFP, [K7Q]GAPR-1-GFP, or ΔMyr-GAPR-1-GFP. At least 100 cells were counted per experiment. The significance of differences was calculated with a two-tailed unpaired t-test (** *p* < 0.01). Scale bar is 2 μm; **C**. MD simulations of non-myristoylated and myristoylated [K7Q]GAPR-1 with on lipid membranes, as described in Fig. 1 (panel D) **D** and **E**. GAPR-1mediated inhibition of autophagy in Hela cells. Stably transfected Hela cells (wtGAPR-1, [K7Q]GAPR-1, or with empty vector (EV)) were incubated with 20 µM Tat-Beclin 1 peptide. After incubation, the number of autophagosomes were analyzed using p62 autophagosome labeling. D) Representative fluorescence images of the stable cell lines showing p62-positive puncta (WT, K7Q, EV from left to right). Scale bar, 10 μm; E) Quantification of p62 puncta per cell (*n* = 3 independent experiments, 27–30 cells per condition per experiment; mean ± SD). Analysis performed using Fiji/ImageJ and GraphPad Prism 10

We previously showed that GAPR-1 interfered with beclin 1 condensate formation, indicating that the protein-protein interaction between these two proteins is based on oligomeric interactions [19]. If [K7Q]GAPR-1 forms different types of oligomers than wtGAPR-1 or [G2A]GAPR-1, as suggested by the co-expression experiments (Fig. 5), then [K7Q]GAPR-1 may not interact with beclin 1 anymore. Indeed, upon co-expression with beclin 1-mCherry, [K7Q]GAPR-1-GFP did not interfere with beclin 1 condensate formation, whereas wtGAPR-1-GFP did reduce the number of cells containing beclin 1 condensates (Fig. 6A and B). The lack of interaction of [K7Q]GAPR-1 with beclin 1 was not due to the concomitant absence of myristoylation, as [G2A]GAPR-1-GFP interfered with beclin 1-mCherry condensates with similar efficiency as wtGAPR-1 (Fig. 6, and as shown previously [16]). These results suggest that although [G2A]GAPR-1 is less efficient in condensate formation, it can form the same type of oligomers as wtGAPR-1, allowing it to interact with beclin 1. Together, these results correlate well with the observed differences in membrane orientation of wtGAPR-1, [G2A]GAPR-1 and [K7Q]GAPR-1 during MD simulations (Fig. 1). Myristoylated wtGAPR-1 has a preferred orientation of 55 degrees (the angle between the N-terminal helix and the vertical axis (relative to the membrane). This presumably allows oligomerization and interaction with beclin 1. Non-myristoylated [G2A]GAPR-1 loses its preferred orientation, but the 55 degree orientation is among the (many) possibilities. Therefore, since [K7Q]GAPR-1 in vivo is also non-myristoylated, our results strongly predict that the lack of myristoylation in the [K7Q]GAPR-1 variant does not result in a loss of a preferred orientation at the membrane, as it does for wtGAPR-1. In addition, if there still is a preferred orientation, it should be different from wtGAPR-1. MD simulations of non-myristoylated [K7Q]GAPR-1 with lipid membranes showed that [K7Q]GAPR-1 does not lose its preferred membrane orientation upon removal of the myristate and obtains a preferred orientation of 75 degrees, which even further deviated from wtGAPR-1 than myristoylated [K7Q]GAPR-1 (Fig. 6C, cf. Figure 1).

To demonstrate the physiological relevance of our MD and yeast overexpression results, we investigated the effect of the K7Q mutation in GAPR-1 in the autophagy process in human cells. Previously we showed that GAPR-1 acts as a negative regulator of autophagy by recruitment of beclin 1 to the Golgi complex [6]. Thus, our results predict that the [K7Q] mutation may also interfere with the capacity of GAPR-1 act as a negative regulator of the autophagic process. We made use of a membrane permeable Tat-beclin peptide (Tat-B18), which specifically interferes with the interaction of GAPR-1 and beclin 1 [6, 19], thus stimulating the autophagic process [6]. Stably transfected Hela cells were incubated with 20 μm Tat-B18 for 1 h to induce autophagy. As a result, relatively few p62-labeled autophagosomes [39] were observed (Fig. 6D, EV). As expected for a negative regulator of autophagy, overexpression of wtGAPR-1 under the same conditions resulted in inhibition of the autophagic process and an accumulation of autophagosomes (Fig. 6D, WT). Interestingly, at similar expression levels, [K7Q]GAPR-1 was significantly hampered in its capacity to act as a negative regulator of autophagy and showed impaired inhibition of the autophagic process (Fig. 6D, K7Q). Together, these results highlight the importance of GAPR-1 myristoylation in enhancing the GAPR-1 orientation that is suitable for oligomeric protein-protein interactions with beclin 1 and subsequent regulation of the autophagic process.

## Discussion

In this study, we identified determinants of the membrane orientation of GAPR-1 at lipid bilayers. Lys7 appears crucial for proper orientation whereas N-terminal myristoylation puts restraints on the motional freedom of GAPR-1 at the membrane. Both mutations that prevent N-terminal myristoylation (G2A and K7Q) strongly affect the capacity of GAPR-1 to form condensates in yeast cells upon overexpression. However, in contrast to [G2A]GAPR-1, [K7Q]GAPR-1 could not affect beclin 1 condensate formation during co-expression anymore. These results indicate that the altered membrane orientation of [K7Q]GAPR-1 is not compatible with the formation of oligomers that are suitable for amyloidogenic interactions with beclin 1.

The lack of condensate formation in the mutant [K7Q]GAPR-1 is not due to a perturbed intrinsic capacity to form amyloid-like aggregates. In vitro, [K7Q]GAPR-1 can form amyloid-like structures and with similar efficiency as wtGAPR-1 (Figs. 3 and 4), indicating that Lys7 does not interfere with conformational changes in the protein required to form cross-beta structures. Then why does [K7Q]GAPR-1 not form biomolecular condensates in yeast upon overexpression? Biomolecular condensate formation starts with the assembly of monomers into liquid-like condensates by phase separation [40–42]. Liquid-like droplets can subsequently harden into a gel-like or glass-like state, which can further convert into solid-like fibrils. Our results therefore suggest that the first step in condensate formation, i.e. the assembly of monomers into oligomers and condensates, is hampered. In these steps, membrane surfaces can provide an essential regulatory platform for the controlled assembly of biomolecular condensates [17, 18, 43]. In vitro, using liposomes or heparin as a concentration platform, this rate-limiting step is bypassed to allow amyloid-like aggregation [10, 44–46]. In vivo, however, the concentration of GAPR-1 monomers and assembly of oligomers on membrane surfaces appears a critical and rate-limiting step in condensate formation. Therefore, our results point to the importance of biological membranes in the assembly of GAPR-1 oligomers.

In MD simulations, the GAPR-1 orientation at negatively charged lipid bilayers is driven by Lys7 and Lys33. To study the effect of these lysines, we mutated Lys7 or Lys33 to glutamine, which neutralizes the charge while mimicking other characteristics of the amino acid side chain. Mutation of K7 to Q (K7Q) or K33 to Q (K33Q) only mildly altered the theoretical isoelectric point (pI) of GAPR-1 from 9.4 to 9.3 (https://web.expasy.org/compute_pi/). The membrane-interacting surface of GAPR-1 that includes the N-terminal myristate group, K7, and K33 is depicted in Suppl. Figure 6. A characteristic lipid binding pocket is missing, but there is some level of specificity in the binding of negatively charged lipids to this surface in MD simulations. The binding affinity decreases in the order PI4P˃PI˃PA˃PS and 4 molecules of PI4P can bind to one GAPR-1 molecule at the same time. These differences in affinity of GAPR-1 for negatively charged lipids as observed by MD remain to be confirmed in vitro and/or in vivo. In vitro, purified recombinant non-myristoylated GAPR-1 binds to negatively charged lipids in liposomes [34] or spotted on nitrocellulose (Suppl. Figure 1), but some differences in relative affinity are observed. We speculate that the restraint in motional freedom of GAPR-1 by myristoylation optimizes the membrane-interacting surface of GAPR-1 (Suppl. Figure 6) and hence the interaction with selected negatively charged lipids. This may be lost in in vitro experiments using non-myristoylated GAPR-1. This is further supported by the absence of a specific lipid binding site, but rather by the presence of a relatively large interaction area to which multiple lipids can bind at the same time, as well as different classes of negatively charged lipids. The significance of different affinities for negatively charged lipids remains to be established, but an interaction with phosphoinositides is interesting and could provide a regulatory mechanism for GAPR-1 membrane binding and oligomerization. Phosphoinositides have multiple functions in cellular physiology, such as serving as second messengers and participating in membrane targeting of proteins [47–49]. Studies have also found that subcellular concentrations of PIP2 mediate the oligomerization behavior of the human serotonin transporter (SERT) [50]. Additionally, PIP2 has been implicated in stabilizing conformation-specific EphA2 dimers, regulating their activation [51]. In this context, it is possible that the lipid environment regulates the oligomerization properties of GAPR-1, providing a regulatory switch to control GAPR-1 dynamics and interactions with other proteins.

Protein myristoylation typically promotes membrane binding that is essential for proper protein localization or biological function [52, 53]. Here, we identified a novel role for protein myristoylation, namely affecting the motional freedom of proteins bound to lipid bilayers. This is interesting in view of the two-signal model for membrane binding of myristoylated proteins: to achieve stable membrane binding, myristoylated proteins require a second membrane binding signal [53]. In the case of GAPR-1, membrane binding is promoted by myristoylation and ionic interactions between lysines and negatively charged lipids. We observed that it depends on the ‘second signal’, i.e. ionic interactions of GAPR-1 with the membrane how myristoylation affects the motional freedom of proteins. In wtGAPR-1, myristoylation restricts the motional freedom, whereas in [K7Q]GAPR-1, in which the second signal has been changed, the orientation is affected but not so much the motional freedom. By affecting the motional freedom ([G2A]GAPR-1), kinetics of protein oligomerization (condensate formation) are affected without affecting the oligomeric properties that allow protein-protein interactions to beclin 1. Thus, the N-myristoyl-group positions GAPR-1 in an optimal orientation to allow rapid oligomerization and interaction with beclin 1.

The specific GAPR-1 orientation at the membrane determines the oligomerization process and an incorrect orientation is not compatible with GAPR-1 oligomerization. [G2A]GAPR-1 co-localizes with wtGAPR-1 condensates upon co-expression, suggesting that [G2A]GAPR-1 can form homo-oligomeric structures with wtGAPR-1 and therefore forms similar amyloidogenic oligomeric structures as the wt protein. In contrast, [K7Q]GAPR-1 does not co-localize with wtGAPR-1 condensates upon overexpression, indicating that its binding/orientation to the membrane is incompatible with the binding of wtGAPR-1. It can form oligomers (and amyloids), but these are distinct oligomeric structures that are not compatible with those of wtGAPR-1. The conclusion that [K7Q]GAPR-1 forms different oligomeric structures as compared to wtGAPR-1 and [G2A]GAPR-1 is strengthened by the observed differences in interaction with beclin 1. [K7Q]GAPR-1 does not affect beclin 1 condensate formation upon co-expression in yeast, in contrast to wtGAPR-1 and [G2A]GAPR-1. We previously showed that the interaction between wtGAPR-1 and beclin 1 is based on amyloidogenic interactions prior to condensate formation [19]. Therefore, our combined results suggest that [K7Q]GAPR-1 forms different oligomeric structures, which is related to the altered orientation at membranes, prior to forming oligomers. Formally, we cannot exclude that Lys7 itself may be directly involved in GAPR-1 dimerization and/or oligomerization as part of the protein-protein interaction interphase, either in homo-oligomerization between GAPR-1 molecules or in hetero-oligomerization with beclin 1. However, this seems unlikely as Lys7 is not present at the dimerization interface of GAPR-1 [5, 8]. Lys7 is also not part of the 5 m GAPR-1 surface predicted by others to interact with beclin 1 [54].

In conclusion, our study adds a new layer of complexity to our understanding of the relationship between GAPR-1, cellular membranes, and oligomerization. It is important to emphasize that there is currently no evidence supporting GAPR-1 amyloid-like aggregate formation in (human) cells. GAPR-1 has a tendency to form dimers and oligomers in mammalian cells also shown by crosslinking experiments [4, 5]. More recently, we showed that GAPR-1 oligomerization is important for its interaction with beclin 1 [19] and that the oligomerization properties of GAPR-1 is based on amyloidogenic interactions. However, we consider it unlikely that GAPR-1 forms amyloid-like aggregates in mammalian cells as (i) the GAPR-1 concentration in cells is very low [4] and (ii) amyloid structures are not reversible, making it very difficult as part of physiological process to regulate autophagy. GAPR-1 does form amyloids in vitro and we use the in vitro aggregation properties of GAPR-1 as a readout to study the amyloidogenic properties involved in GAPR-1 oligomerization, which are relevant for its function. The identification of the K7Q mutation emphasizes this issue by showing that whereas it does not inhibit GAPR-1 amyloid formation in vitro, it does affect the interaction with beclin 1 and the autophagy process. Along similar lines we note that the condensate formation of GAPR-1 is likely a result of the overexpression system in yeast and it is not clear whether biomolecular condensates play a role in its physiological function. Nevertheless, the same amyloidogenic interactions that play a role in the formation of these condensates, do play a role in GAPR-1 oligomerization in lipid-enriched microdomains t the Golgi complex and its interaction with beclin 1.

In summary, our results may hint to novel ways to interfere with amyloidogenic oligomer formation of GAPR-1. GAPR-1 membrane binding and interaction with negatively charged lipids can serve as a switch to regulate the amyloidogenic assembly of GAPR-1 oligomers and hence, oligomeric protein-protein interactions with other proteins such as beclin 1. Further exploration of these interactions may unveil novel insights into the functional consequences of GAPR-1 behavior and its potential implications in cellular homeostasis and disease.

## Supplementary Information

Below is the link to the electronic supplementary material.

Supplementary Material 1 (DOCX 2.88 MB)

## Data Availability

The datasets generated during the current study are available from the corresponding author on reasonable request.
