# Supplementary material for: Membrane orientation regulates GAPR-1 oligomerization that is suitable for interaction with beclin 1
Source: Cell Mol Life Sci. 2026 Jul 28;83(1):392. doi: 10.1007/s00018-026-06329-4 (PMC13690689; doi:10.1007/s00018-026-06329-4)
Supplement: Supplementary file 1 [file 18_2026_6329_MOESM1_ESM.docx]

**Membrane orientation regulates GAPR-1 oligomerization**

**that is suitable for interaction with beclin 1**

Ziying Shen^1^, Robin A. Corey^2^, Hidde van Doorne^1^, Iris Verhoek^1^, Renée Koopman^3^, Daniel S. Huitema^4^, Samiksha Sardana^4^, Marc P. Baggelaar^4^, Phillip J. Stansfeld^5^, Dora V. Kaloyanova^1^, J. Bernd Helms^1*^

^1^Division of Cell Biology, Metabolism and Cancer, Faculty of Veterinary Medicine, Utrecht University, Utrecht, the Netherlands

^2^School of Physiology, Pharmacology & Neuroscience, Biomedical Sciences Building University Walk, Bristol, BS8 1TD, United Kingdom

^3^Protein Research Centre, Department of Chemistry, Faculty of Science, Utrecht University, Utrecht, the Netherlands

^4^Biomolecular Mass Spectrometry & Proteomics, Faculty of Science, Utrecht University, Utrecht, the Netherlands

^5^School of Life Sciences and Department of Chemistry, University of Warwick, Coventry, CV4 7AL, United Kingdom

Key words: *CAP superfamily of proteins, protein-lipid interactions, protein-protein interactions, protein oligomerization, N-myristoylation, autophagy??*

^*^To whom correspondence should be addressed: J.B.H. ([J.B.Helms@uu.nl](mailto:J.B.Helms@uu.nl))

**Shen et al. Supplementary Figures and Legends:**

**Supplementary Figure 1. WT GAPR-1 phosphoinositide affinity**

A. Recombinant non-myristoylated wtGAPR-1 protein (0.5 µg/ml in NT-50 buffer) was incubated with PIP strip membranes containing different lipids as indicated (100 pmol/spot). Bound GAPR-1 was detected using anti-GAPR-1 antibodies as described in Materials and Methods;

B. Quantification of the number of lipids bound to monomeric GAPR-1 during CG-MD simulation of GAPR-1 interactions with lipid bilayers consisting of PC/PE/PS/PIP (50/30/10/10). 4 PI4P molecules bind to monomeric GAPR-1 without significant PS binding.

**Supplementary Figure 2. Expression levels of GAPR-1 in Saccharomyces cerevisiae**

Western blot analysis of GAPR-1-GFP levels in yeast following overexpression of wtGAPR-1-GFP and [K7Q]GAPR-1-GFP. Equal amounts of total protein lysate were separated by SDS-PAGE and analyzed by Western blotting using an anti–GAPR-1 antibody.

**Supplementary Figure 3. GAPR-1 is not palmitoylated**.

A. Acyl-biotin exchange (ABE) analysis of Caveolin and GAPR-1. Western blot of ABE-enriched fractions obtained in the presence (+HA) or absence (−HA) of hydroxylamine, alongside corresponding total non-enriched proteome lysate inputs. Caveolin, used as positive control for S-acylation, was detected exclusively in the +HA fraction and was absent in the −HA control, confirming hydroxylamine-dependent enrichment. GAPR-1 was not detected in either +HA or −HA ABE-enriched fractions, despite comparable expression levels in the lysate inputs, pointing towards GAPR-1 not being S-palmitoylated. B. Representative live cell fluorescence microscopy images of yeast cells after 18 h of expression of wtGAPR-1-GFP or [C32S]GAPR-1-GFP as indicated. Scale bar is 2 µm; Quantification of yeast cells displaying GAPR-1-GFP condensates upon expression of wtGAPR-1-GFP and [C32S]GAPR-1-GFP in approximately 100 cells per condition per experiment, analyzed in three independent experiments. Data are shown as mean ± SD.

**Supplementary Figure 4. Role of K7 in GAPR-1 binding to liposomes**

A. Dot blot detection of recombinant non-myristoylated GAPR-1(WT/K7Q) purified protein amounts before incubation with liposomes. Increasing amounts of GAPR-1 (0.125-16 µg) are spotted on nitrocellulose membranes and GAPR-1 was detected using anti-GAPR-1 antibodies as described in Materials and Methods;

B. Representative fluorescence microscopy images from incubations at 37 ^o^C for 1 day as indicated and consisting of purified recombinant non-myristoylated 2 µM GAPR-1 protein with 200 mM liposomes in the presence of low salt (50 mM) or high salt (200 mM). WT: wtGAPR-1-GFP; K7Q: [K7Q]GAPR-1-GFP; +PC: PC liposomes (100%PC); +10%PI: PC/PI (90%/10%) liposomes; NT-50: NT-50 buffer containing 50 mM NaCl; N200: NT buffer containing 200 mM NaCl. Scale bar is 2 µm.

**Supplementary Figure 5. Thermal stability of wtGAPR-1 and [K7Q]GAPR-1**

Thermal and chemical stability of GAPR-1(WT/K7Q) was analyzed by Differential Scanning Fluorimetry (nanoDSF) (Prometheus Panta, NanoTemper Technologies, Germany). Concurrent changes in tryptophan fluorescence at 330 and 350 nm wavelength were monitored, whereby the ratio 350 nm/330 nm reflects GAPR-1 conformational changes under different temperatures (35-65 ℃).

A. WT/K7Q GAPR-1 protein in NT-50 buffer;

B. WT/K7Q GAPR-1 incubated with 37.5 μM heparin in NT-50 buffer;

C. WT/K7Q GAPR-1 protein incubated with 100 μM Zn^2+^ in NT-50 buffer;

D. WT/K7Q GAPR-1 protein incubated with 37.5 μM heparin and 100 μM Zn^2+^ in NT-50 buffer.

**Supplementary Figure 6. GAPR-1 membrane interaction area**

The membrane-interacting surface of GAPR-1 was determined after completion of the MD simulations. Red color represents acidic residues; blue color represents basic residues; green color represents polar residues; white represents hydrophobic residues; yellow spheres represents the myristoyl-group (pointing perpendicular to area).
